# Inhibition of AMD-Like Pathology With a Neurotrophic Compound in Aged Rats and 3xTg-AD Mice

**DOI:** 10.3389/fnagi.2019.00309

**Published:** 2019-11-19

**Authors:** Yinghua Liu, Wei Wei, Narjes Baazaoui, Fei Liu, Khalid Iqbal

**Affiliations:** ^1^Department of Neurochemistry, Inge Grundke-Iqbal Research Floor, New York State Institute for Basic Research in Developmental Disabilities, Staten Island, NY, United States; ^2^Department of Pharmacology, School of Pharmaceutical Sciences, Guangzhou Medical University, Guangzhou, China; ^3^Key Laboratory of Molecular Clinical Pharmacology, Fifth Affiliated Hospital, Guangzhou Medical University, Guangzhou, China; ^4^Key Laboratory of State Administration of Traditional Chinese Medicine of China, Department of Pathophysiology, School of Medicine, Institute of Brain Research, Jinan University, Guangzhou, China

**Keywords:** age-related macular degeneration, Alzheimer’s disease, retina, neurotrophic peptidergic compound P021, prevention of AMD, aged rats, 3xTg-mice

## Abstract

Age-associated macular degeneration (AMD), which leads to loss of vision at its end stage, is one of the most common neurodegenerative diseases among the elderly. However, to date, no effective drug therapy is available for the prevention of AMD. Here, we report the occurrence of AMD pathology and its prevention by chronic treatment with the neurotrophic peptidergic compound P021, in aged rats and 3xTg-AD mice. We found photoreceptor degeneration, lipofuscin granules, vacuoles, and atrophy in retinal pigment epithelium (RPE) as well as Bruch’s membrane (BM) thickening; in aged rats, we even found rosette-like structure formation. Microgliosis and astrogliosis were observed in different retinal layers. In addition, we also found that total tau, phosphorylated tau, Aβ/APP, and VEGF were widely distributed in the sub-retina of aged rats and 3xTg mice. Importantly, chronic treatment with P021 for 3 months in rats and for 18 months in 3xTg mice ameliorated the pathological changes above. These findings indicate the therapeutic potential of P021 for prevention and treatment of AMD and retinal changes associated with aging and Alzheimer’s disease.

## Introduction

Age-associated macular degeneration (AMD) involves the degeneration of the small central area of the retina, called macula, that controls visual acuity. AMD is a leading cause of vision loss in the elderly. In AMD, the macula is irreversibly destroyed, which leads to loss of vision required for activities such as driving, recognizing faces, reading, and seeing in color (Lambert et al., 2016).

The prevalence of AMD increases with age. The risk of getting AMD increases from 2% at 50–59 years of age to almost 30% at 75 years of age. The number of AMD cases is estimated to reach 196 million worldwide by 2020 and 288 million by 2040 if no effective treatment is developed (Wong et al., 2014).

There are two forms of AMD: a dry form and a wet form. The dry form, which is most common, is non-neovascular. It leads to atrophy of the retinal pigment epithelium (RPE), choriocapillaris, and photoreceptors. The wet AMD is neovascular. It occurs when new blood vessels invade from the choroid and penetrate Bruch’s membrane (BM), causing vascular leakage, hemorrhage, and scarring. Wet AMD is less common than the dry form but choroidal neovascularization (CNV) in this form leads to vision loss. Dry AMD is believed to result from the aging and thinning of macular tissues, deposition of pigment in the macula, or a combination of these two processes (Bhutto and Lutty, 2012).

AMD is a multifactorial disease, the etiopathogenesis of which probably involves several factors that are currently not understood (Lambert et al., 2016). A growing body of evidence suggests that the immune system plays a key role in triggering neuroinflammation in the retina in AMD development (Ambati et al., 2013). There are no early biomarkers to anticipate AMD, and no FDA-approved treatments are available, although a few are in clinical trials, and nutritional intervention may help prevent its progression from the dry to the wet form (Bandello et al., 2017).

The retina is a part of the CNS and offers a direct window to cerebral pathology. Like brain, retina contains neurons, astroglia, microglia and microvasculature and participates in similar physiological activities and shows some of the similar neurodegenerative changes that have been reported in Alzheimer’s disease (AD; Morin et al., 1993; Patton et al., 2005; Hart et al., 2016; Li et al., 2016; Trost et al., 2016; Vecino et al., 2016; Koronyo et al., 2017). The development of ocular biomarkers can potentially help AD drug discovery (Lim et al., 2016). AMD and AD share several similarities which include neurodegeneration, neuroinflammation, and accumulation of β-amyloid (Aβ) and hyperphosphorylated tau (Johnson et al., 2002; Ohno-Matsui, 2011; Frost et al., 2016). AMD has also been referred to as AD of the eye (Kaarniranta et al., 2011).

P021 is a small peptidergic compound derived from ciliary neurotrophic factor (CNTF) that is orally bioavailable and is blood-brain barrier (BBB)–permeable; it enhances dentate gyrus neurogenesis and neuronal plasticity by competitively inhibiting the leukemia inhibitory factor and by increasing the expression of brain-derived neurotrophic factor (BDNF; Blanchard et al., 2010; Li et al., 2010; Chohan et al., 2011; Kazim et al., 2014). In previous studies, both P021 and its parent non-adamantylated peptide were found to rescue cognitive impairment, synaptic deficit, neuroinflammation, and tau and Aβ pathologies in rat and mouse models of AD (Blanchard et al., 2010; Chohan et al., 2011; Bolognin et al., 2014; Kazim et al., 2014; Baazaoui and Iqbal, 2017a,b).

Despite lacking a macula, the retina of old mice shows many AMD features and has been useful in studying risk factors for AMD, including environment, age, genetics, diet, smoking, and inflammation (Espinosa-Heidmann et al., 2006; Handa, 2012; Pennesi et al., 2012; Lambert et al., 2016). Several animal models of AMD have been generated and have enhanced understanding of the etiopathogenesis of the disease (Pennesi et al., 2012). Here, we describe the AMD-like pathology in aged rats and mice, and the beneficial effect of P021 on AMD and retinal changes associated with aging and AD in these models.

## Materials and Methods

### Antibodies and Reagents

The primary antibodies used in this study are listed in Table 1. Alexa Fluor 488-conjugated goat anti-mouse and Alexa Fluor 488-conjugated goat anti-rabbit IgG, TO-PRO^TM^-3 Iodide, and ProLong Gold Anti-fade reagent were obtained from Thermo Fisher Scientific (Rockford, IL, USA). Other chemicals were from Sigma-Aldrich (St. Louis, MO, USA).

**Table 1:** Primary antibodies used in this study.

Antibody	Specificity	Species	Type	Dilution	Source/Reference	Cat. No.
Iba-1	Iba-1	R	Poly-	1:1,000	Wako Pure Chemical Industries Limited (Richmond, VA, USA)	019-19741
SMI22	GFAP	M	Mono-	1:2,000	Sternberger Monoclonals Inc. (Lutherville, MD, USA)	SMI22
R134d	Total tau	R	Poly-	1:1,000	Grundke-Iqbal et al. (1988)
PHF-1	P-tau (Ser396/404)	M	Mono-	1:200	Greenberg et al. (1992)	Gifted by Dr. Peter Davies
AT-8	P-tau (Ser202/T205)	M	Mono-	1:1,000	Thermo Fisher Scientific (Rockford, IL, USA)	MN1020
4G8	Aβ/APP	M	Mono-	1:1,000	Biolegend (San Diego, CA, USA)	800712
Anti-A/β	Aβ/APP	R	Mono-	1:500	Cell Signaling Technology (Danvers, MA, USA)	14974
Anti-VEGF (C-1)	VEGF	M	Mono-	1:100	Santa Cruz Biotechnology (Dallas, TX, USA)	sc-7269

*Key: Iba-1, ionized calcium-binding adaptor molecule 1; GFAP, glial fibrillary acidic protein; VEGF, vascular endothelial growth factor; p-tau, phosphorylated tau; APP, amyloid precursor protein; Aβ, amyloid-β; Poly-, polyclonal; Mono-, monoclonal; R, rabbit; M, mouse*.

### Synthesis and Features of P021

The peptidergic compound P021 (Ac-DGGL^A^G-NH2; mol. wt. of 578.3) corresponds to a biologically active region of human CNTF (amino acid residues 148–151) to which adamantylated glycine was added at the C-terminal to increase its stability and lipophilicity (Blanchard et al., 2010; Li et al., 2010; Chohan et al., 2011). The peptide was synthesized and purified by reverse-phase high-performance liquid chromatography to ~96% purity, and the sequence of the peptide was confirmed by mass spectrometry, as described previously (Li et al., 2010).

P021 is quite stable in artificial gastric juice (~90% for 30 min) and in artificial intestinal juice (>95% for 120 min). BBB studies on P021, which were carried out through a commercial service (APREDICA, Watertown, MA, USA), demonstrated that a sufficient amount of P021 crossed the BBB to exert its effect in the brain (Kazim et al., 2014).

### Animals

Female Fisher Rats ~19–21 months and ~2–3 months of age (Charles River, France) weighing approximately 300 g were used. Fisher rats are commonly used for studies on aging, and the aged animals are commercially available. Unlike males, which continue to add weight, female rats present a relatively stable weight of ~300 g from ~3 months to 24 months, and we successfully used these animals for our previous study, in which we investigated the chronic effect of compound P021 on cognitive aging (Bolognin et al., 2014). The vehicle-treated 2- to 3-month-old rats were used as a reference to study AMD-like pathology in the aged animals and the magnitude of the rescue of these changes by P021 treatment.

Homozygous 3xTg-AD mice harboring human APP_SWE_ and tau_P301L_ transgenes with knock-in PS1_M146V_ under the control of the mouse Thy1.2 promoter, generated in the laboratory of Dr. Frank LaFerla (Oddo et al., 2003), and age-matched control mice of the same genetic background (hybrid 129/Sv×C57BL/6) were obtained from the Jackson Laboratory^1^. Male and female 3xTg-AD mice and age-matched control mice were bred in the Laboratory Animal Resource Center of the New York State Institute for Basic Research in Developmental Disabilities (Staten Island, NY, USA).

Animals were housed at a standard temperature (22 ± 1°C) and in a light-controlled environment (lights on from 7 AM to 8 PM), had access to food and water *ad libitum*, and were housed (four or five animals per cage) in pathogen-free facilities with 12-h light/12-h dark cycles. All animal studies were carried out according to the National Institutes of Health guidelines for the care and use of laboratory animals. Oral treatment of rats with P021 or vehicle was carried out as a paid service at Charles River Labs, Finland, and was approved by the National Animal Experiment Board, Finland.

### Animal Treatment

**Figure 1:**
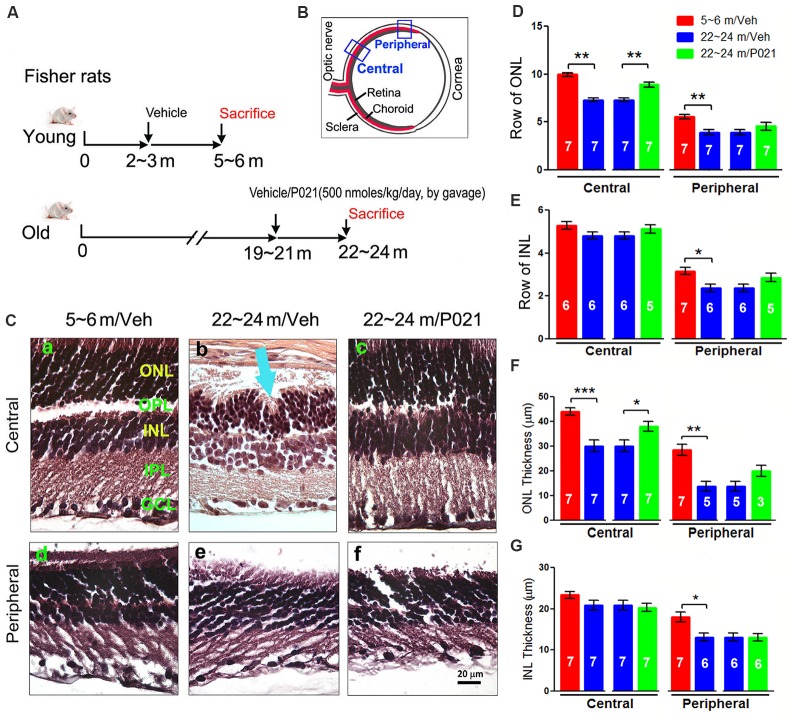
AMD-like retinal pathology is reduced by P021 treatment in aged rats. **(A)** Experimental design to study the effect of P021 treatment on retina in aged rats. Fisher rats at age ~19–21 months received P021 in saline (500 nmoles/kg body weight/day, by gavage) or vehicle only for 3 months, whereas ~2- to 3-month-old rats received vehicle only for 3 months. **(B)** Schematic diagram showing the sections of the rat eye employed in the study. **(C)** Representative images of the central and peripheral retinas showing the morphology of retinal layers, H & E staining in 5-μm sagittal paraffin sections **(Ca–f)**. Rosette-like rearrangements of photoreceptor cells that extend from the ONL and across the OPL toward INL **(Cb)**, and photoreceptor inner segments (ISs) that project inward to form the core of the rosette (blue arrow) were found in ~22- to 24-month-old vehicle rats. **(D–G)** Quantification of rows and thickness of the ONL and INL in central and peripheral retina (*n* = 3–7 rats; exact “*n*” values are labeled in each bar). The Student’s *t*-test was used to analyze the data between 5–6 m/Veh and 22–24 m/Veh, and between 22–24 m/Veh and 22–24 m/P021 rats. The data are shown as mean ± SEM. *P < 0.05, **P < 0.01, ***P < 0.001. AMD, age-related macular degeneration; ONL, outer nuclear layer (photoreceptor cell bodies); OPL, outer plexiform layer; INL, inner nuclear layer; IPL, inner plexiform layer; GCL, ganglion cell layer; m, months; H&E, hematoxylin and eosin.

Female aged (19–21 months) Fisher rats (*n* = 7) were given P021 *per os* by gavage (10 ml/kg body weight) once a day for 88 days. The dose of P021 was 500 nanomoles (289.15 μg)/kg body weight daily; the dose was based on our previous study, in which we found that this dose could rescue cognitive impairment in aged Fisher rats (Bolognin et al., 2014). To be able to deliver the same constant dose to each animal, we chose gavage. Furthermore, the administration of P021 by gavage is more economical than by drinking water. As controls, the second group of aged (19–21 months) female rats (*n* = 7) and a group of young adult female rats (2–3 months, *n* = 7) were treated identically, but with normal saline (vehicle) only (Figure 1A). Administration of vehicle and test compound was done at 7–9 AM daily and blindly to the investigators who collected, processed, and analyzed the tissue.

At approximately 3 months of age, the female wild type and 3xTg-AD mice were divided into five groups (*n* = 6–7 mice per group; Figure 3A): (1) wild type mice, age 3 months, without treatment (WT-3 m); (2) 3xTg-AD mice, age 3 months, without treatment (3xTg-3 m); (3) wild type mice, age 3 months, treated with vehicle feed without P021 until the age of 21 months (WT-21 m/Veh); (4) 3xTg-AD-mice, age 3 months, treated with vehicle feed without P021 until the age of 21 months (3xTg-21 m/Veh); and (5) 3xTg-AD-mice, age 3 months, treated with 60 nmol P021/g feed till the age of 21 months (3xTg-21 m/P021). The first two groups, WT-3 m and 3xTg-AD-3 m, were the starting points and were employed as controls to study age-associated AMD-like changes in WT-21 m and 3xTg-AD-21 m, respectively. We included WT-21 m/Veh as another reference group to enable a comparison of AMD-like changes in 3xTg-21 m/Veh with age-matched WT animals. Given that the study in Fisher rats demonstrated the therapeutic effect of P021 in the aged animals, we did not include the WT-21 m/P021 mice group because the investigation of the genotype effect was not our objective in the mouse study. P021 was formulated in the feed by Research Diets (New Brunswick, NJ, USA). Food consumption and body weight were recorded every 2 weeks and every month, respectively. The average mouse food consumption was ~2.7 g feed/day. The analysis of the animals was carried out blind to the treatment.

**Figure 2:**
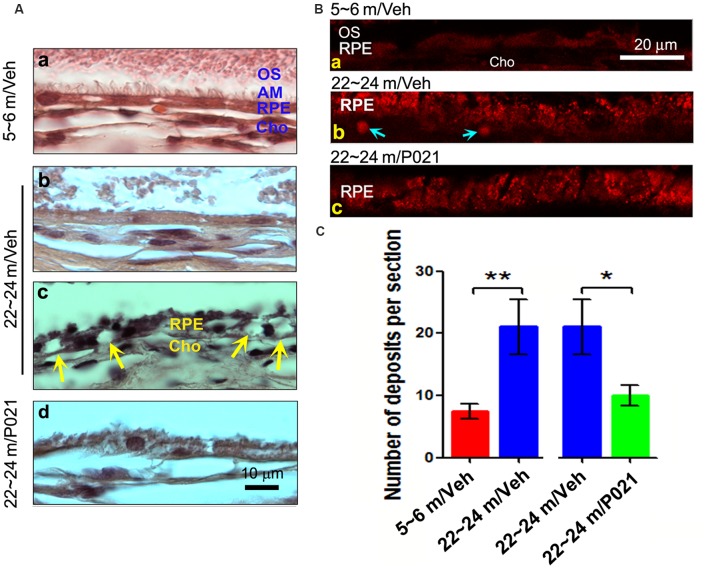
Chronic treatment with P021 reduces AMD-like pathology of RPE in aged rats. **(A)** H & E staining of RPE in the retina. AM was short or out of order, and atrophy and vacuolization of RPE (**Ac**, yellow arrow) were found in ~22- to 24-month-old/vehicle rats; no vacuolization was detected in other animal groups **(Aa,b,d)**. **(B)** Representative images of auto-fluorescence at λ_ex_ = 543 (red channel). The number of deposits between RPE and Cho (**Bb**, blue arrow), and lipofuscin granules were markedly greater in ~22- to 24-month-old/vehicle rats than in ~5- to 6-month-old/vehicle rats **(Ba)** or ~22- to 24-month-old/P021 rats **(Bb,c)**. **(C)** Quantification of deposits number (*n* = 7 rats). Students, *t*-test was used to analyze the data between 5–6 m/Veh and 22–24 m/Veh, and between 22–24 m/Veh and 22–24 m/P021 rats. The data are shown as mean ± SEM. *P < 0.05, **P < 0.01. RPE, retinal pigment epithelium; AM, apical microvilli; BM, Bruch’s membrane; Cho, choroid; OS, outer segment.

**Figure 3:**
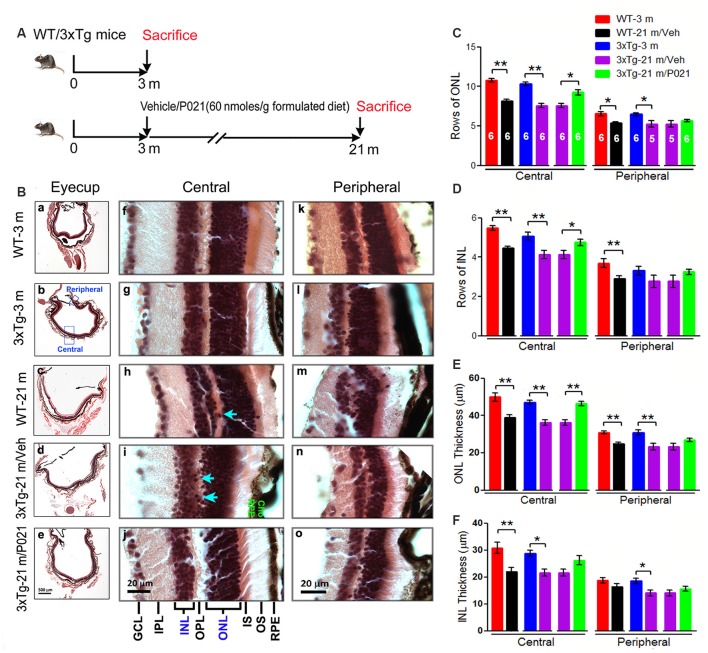
AMD-like retinal pathology is prevented by P021 treatment in aged 3xTg-AD mice. **(A)** Experimental design to study the effect of chronic treatment with P021 on retina in aged 3xTg-AD mice. Starting at 3 months of age, 3xTg mice received mouse chow (AIN-76, Research Diets, New Brunswick, NJ, USA) without (vehicle) or containing P021 (60 nmoles/g diet); and WT animals received only vehicle diet. At 21 months of age, they were perfused with 0.1 M phosphate-buffered saline (PBS). A second group of WT and 3xTg-AD mice at 3 months of age was employed as young controls. **(B)** H & E staining to detect retinal morphology in 5-μm-thick sagittal paraffin sections; **(a–e)**. The sagittal eyecup sections parallel to the axis from pupil to optic nerve; **(b)**. Position of central and peripheral retinas are labeled in blue; **(f–j)**. Representative images of central retina; **(h,i)**. The extension of cell nuclei from the ONL to the INL (blue arrow); disruption and atrophy of RPE with increased brown lipofuscin granules (**i** green “RPE”); **(k–o)**. Representative images of peripheral retina. **(C–F)** Quantification of rows and thickness of the ONL and INL in the central and peripheral retina (*n* = 5–6 mice****; exact “*n*” values are labeled in each bar of panel **(C)**. Students, *t*-test was used to analyze the data between WT-3 m and WT-21 m/Veh, between 3xTg-3 m and 3xTg-21m/Veh, and between 3xTg-21m/Veh and 3xTg-21 m/P021 mice. The data are shown as mean ± SEM. *P < 0.05, **P < 0.01. AMD, age-related macular degeneration; WT, wild type; 3xTg, triple-transgenic; AD, Alzheimer’s disease; RPE, retinal pigment epithelium; OS, outer segment; IS, inner segment; ONL, outer nuclear layer; OPL, outer plexiform layer; INL, inner nuclear layer; IPL, inner plexiform layer; GCL, ganglion cell layer. NFL, nerve fiber layer.

### Tissue Processing

The animals were anesthetized by using an overdose of avertin and then were transcardially perfused by using 0.1 M phosphate-buffered saline (PBS). Eyeballs were dissected out from the carcasses, immersion-fixed for 24 h in 4% paraformaldehyde at 4°C, and then transferred to 70% alcohol for storage; in the case of mice, the eyeballs were prefixed by injecting 4% paraformaldehyde, followed by removal from the carcasses and immersion fixation as above. After the cornea and lens of the left eyes were removed, the remaining eye cup was embedded in paraffin, with the orientation parallel to the optic nerve in longitudinal position. In all cases, 5-μm serial sections were cut by using a rotary microtome and were mounted on Superfrost Plus slides. Processing and analysis of the retinal tissue were conducted blind.

### Histological Analysis

Every 25th retinal section was deparaffinized and subjected to hematoxylin and eosin (H & E) staining to evaluate the morphology. The H & E staining images were captured with a light microscope using 2×, 40×, and 100× objective lenses. Because there is no significant difference between the 1/5th to the 1/3rd of the retina from the optic nerve head and the most peripheral part, we chose the 1/5th position of the retina from the optic nerve to the most peripheral part of the retina as the “central,” and the most peripheral part of the retina as “peripheral.” Rows of the outer nuclear layer (ONL) and the inner nuclear layer (INL) of the retina in each group (every 25th section, 3–5 sections per rat or mouse, *n* = 5–7 rats per group; *n* = 5–6 mice per group) were manually counted in three columns per image by using a 40× or, where necessary, a higher magnification objective. The thicknesses of the ONL and INL of the central and peripheral retina in each group (every 25th section from the optic nerve head, 2–3 sections per rat or mouse, *n* = 3–7 rats per group; *n* = 5–6 mice per group) were measured and quantified by using the ImageJ/NIH image analysis system. Processing and analysis of the retinal tissue and the data were conducted blind to the genotypes and the treatment of the animals.

### Autofluorescence

Paraffin sections were deparaffinized, rehydrated, and covered with coverslips with 0.1 M PBS and then photographed by using a confocal microscope with a red channel (λ_ex_ = 543, λ_em_ = 590/50), as described previously (Marmorstein et al., 2002). To analyze the lipofuscin granules in RPE (Anderson et al., 2002; Radu et al., 2005) and the deposits between RPE and choroid (Cho; Marmorstein et al., 2002), the sections were photographed by using a 60× objective lens. For quantification, the number of deposits between RPE and Cho in each section was manually counted by using a 20× or, where necessary, a higher magnification objective.

### Immunofluorescence

Paraffin sections were deparaffinized, rehydrated, and subjected to antigen retrieval by boiling for 20 min in 10 mM sodium citrate solution (pH 6.0). The sections were then washed three times in 10 mM PBS for 15 min each and incubated in 0.3% Triton X-100 for 30 min. The sections were again washed in 10 mM PBS (15 min, three times each) and blocked in blocking solution (5% normal goat serum containing 0.1% Triton X-100 and 0.05% Tween 20 in PBS) for 45 min. The sections were then incubated overnight at 4°C with the corresponding primary antibodies (see Table 1 for antibodies used in this study) diluted in the same blocking solution. After being washed three times for 15 min each with 10 mM PBS, sections were incubated with Alexa-Fluor 488-conjugated goat anti-mouse or anti-rabbit IgG secondary antibodies (1:1,000) in 10 mM PBS with 0.05% Tween 20 for 2 h at room temperature. Sections were subsequently washed one time and incubated with To-Pro (1:1,000) for 15 min. After being washed three times for 15 min, sections were mounted and cover-slipped by using ProLong Gold anti-fade reagent. The absence of primary antibody control staining was included in each experiment as a negative control. Double immunofluorescence and To-Pro images were captured at equal camera exposure (for each antibody staining) with a Nikon EZ-C1 laser confocal imaging system. The area to be analyzed was selected, converted to grayscale, and thresholded using Yen’s arithmetic, and the area percentage of positive staining was measured for each section by using the NIH ImageJ software package. The staining area was quantified from two to three sections of each eye and averaged (*n* = 5–7 animals per group).

### Statistical Analysis

Data were analyzed using Prism version 5.0 software (Graph Pad Software Inc., La Jolla, CA, USA). Based on the experimental design, *t*-test was used to compare the data between 5–6 m Veh and 22–24 m Veh rats, between 22–24 m Veh and 22–24 m P021 rats, or between WT-3 m/Veh and WT-21 m/Veh mice, between 3xTg-3 m/Veh and 3xTg-21 m/Veh mice, and 3xTg-21 m/Veh and 3xTg-21 m/P021 mice. All data were computed as mean ± SEM. *P* < 0.05 was considered statistically significant. This study was limited to investigation of AMD-like changes in aged rats and mice, and to the prevention of these changes in aged rats and 3xTg-AD mice by chronic treatment with P021, and did not include WT mice treated with P021 group. We, therefore, could not analyze the data with two-way ANOVA to separate age and genotype effects. The effect of age and the effect of P021 treatment were therefore analyzed separately by the Student’s *t*-test. Since AMD-like changes are seen in old and not young mice, no statistical comparison between 3-month-old WT and 3-month-old 3xTg-AD mice was made. The analysis of data was performed by someone blind to the genotypes of the animals and the test reagent.

## Results

### P021 Rescues the Retinal Pathology in Aged Fisher Rats

At as early as 12 months of age, OXYS rats and Wistar rats are known to show AMD-like pathology, such as BM thickening and lipofuscin accumulation (Neroev et al., 2008; Markovets et al., 2011). When we previously evaluated the effect of chronic oral treatment with P021 on tau pathology and cognitive impairment in aged rats and mice, we did not observe any worsening in general physical state because of P021 treatment, suggesting a probable lack of any side effects (Kazim et al., 2014). Here, to assess the AMD-like retinal pathology induced by aging and to study the effect of P021 on the pathology, we investigated AMD features in rats. Young (~2–3 months of age) and aged (~19–21 m) female Fisher rats were chosen for this study. The female Fisher rats offer a relatively stable body weight of ~300 g from the age of ~3 months to ~24 months, and the aged animals are commercially available. Briefly, the aged rats were administered P021 (500 nmoles/kg/day) in saline or vehicle only by gavage, and the young rats were treated with vehicle only for 3 months (Figure 1A). We studied the morphological changes in sections of central and peripheral retinas after 3 months’ treatment (Figures 1B,C). Photoreceptor cell loss was evaluated by analyzing the rows and thickness of the layers of photoreceptor cell nuclei (ONL). H & E staining showed that rows and thickness of ONL in the central and peripheral retinas were dramatically decreased in ~22- to 24-month-old rats (Figures 1C,D,F). P021 prevented these pathological changes more effectively in the central retina (Figures 1D,F) than in the peripheral retina (Figures 3D,F). There was also no significant difference between ~5- to 6-month-old rats and P021-treated ~22- to 24-month-old rats (Figures 1D,F), which suggests that P021 can prevent ONL lesions. Also, the rows and thickness of INL in the peripheral retina were decreased in the ~22- to 24-month-old/Veh rats compared with the ~5- to 6-month-old/Veh rats, and ~22- to 24- month-old/P021 rats showed a clear trend to rescue the number of rows (Figures 1C,E,G). There were no significant differences in the rows and thickness of INL in the central retina between ~5- to 6-month-old/Veh and ~22- to 24-month-old/Veh rats, and P021 had no detectable effect (Figures 1C,E,G). Additionally, we found that the laminar arrangement in the central retina in ~22- to 24-month-old/Veh rats was distorted by outer retinal folds and rosette-like structures (Figure 1Cb). These aberrations involved a semi-spherical organization of photoreceptor cell nuclei that occupied the ONL, extended across the outer plexiform layer (OPL), and encroached into the INL. The center of the rosettes appeared to be occupied by the photoreceptor inner segment (IS). No abnormal structure like this was observed in the ~5- to 6-month-old/Veh rats or ~22- to 24-month-old/P021 rats (Figures 1Ca,c,d,f). Altogether, these results indicated photoreceptor cell degeneration in the central retina, and INL degeneration in the peripheral retina in ~22- to 24-month-old/Veh rats, and suggested that 3-month-long treatment with P021 protected ~19- to 21-month-old rats against AMD-like pathology in the central retina.

### P021 Rescues the Pathophysiology of RPE and BM in Aged Rats

Dysfunction of the RPE presages photoreceptor cell loss, and the histological abnormalities in RPE are also a hallmark of human dry AMD (Markovets et al., 2011; Zanzottera et al., 2015). To investigate the histopathological changes in rats, the RPE layer was examined in sagittal sections stained with H & E. Apical microvilli (AM) in RPE were apparent in ~5- to 6-month-old/Veh rats (Figure 2Aa); in contrast, they were shortened or disorganized in the ~22- to 24-month-old/Veh rats (Figures 2Ab–d), and P021 treatment showed improvement in the arrangement of AM. Additionally, many RPE cells were vacuolated (Figure 2Ac, yellow arrows), and RPE atrophy (Figure 2Ac, yellow RPE) was detected in aged rats, whereas no apparent abnormalities were observed in the RPE of ~5- to 6-month-old/Veh rats and ~22- to 24-month-old/P021 rats (Figures 2Aa,d). Furthermore, many lipofuscin granules (Figures 2Ac,Bb) and deposits (Figures 2Bb,C) were present between RPE and Cho detected by auto-fluorescence in ~22- to 24-month-old/Veh rats, whereas very few lipofuscin granules were detected in ~5- to 6-month-old/Veh rats (Figures 2A,Ba); lipofuscin granules and deposition number were markedly decreased in ~22- to 24-month-old/P021 rats (Figures 2Bc,C). These results suggest that ~22- to 24-month-old/Veh rats exhibit RPE abnormalities, and P021 ameliorates these pathological changes.

### Chronic Treatment With P021 Can Prevent Retinal Lesions in Aged Mice

AMD-like pathology was reported previously in Tg2576-mice and 5xFAD mice (Liu et al., 2009; Park et al., 2017). The occurrence of AMD-like changes in 3xTg-AD mice (3xTg-AD) was not known. Here, we investigated the presence of AMD-like pathology and its prevention by P021 treatment in aged 3xTg-AD mice.

To evaluate the effect of P021 on the development of retinal pathology, we fed 3-month-old female 3xTg-AD mice with formulated feed containing P021 (60 nmoles/g feed) or vehicle feed until they reached 21 months of age. We used WT-3 m and 3xTg-3 m mice without any treatment as young controls (Figure 3A). Paraffin sections of the eyecup parallel to the axis of the pupil to the optic nerve were employed to study changes in the retina (Figures 3B,a–e). We found that rows and thickness of ONL were all significantly decreased in the central retina in WT-21 m/Veh and 3xTg-21 m/Veh mice and that these changes in the central retina were prevented in 3xTg-21 m/P021 mice (Figures 3B,C,E). In the peripheral retina there was a significant decrease in rows and thickness of ONL in WT-21 m/Veh and only in the thickness but not in rows of ONL in 3xTg-AD-21 m/Veh, and P021 treatment showed a trend to rescue these changes (Figures 3C,E). Rows and thickness of INL in the central retina were also decreased in WT-21 m/Veh and 3xTg-21 m/Veh mice, and chronic treatment with P021 prevented these changes in 3xTg-21 m mice; although there was an obvious trend, the rescue of INL thickness by P021 treatment did not reach statistical significance (Figures 3B,D,F). The age-associated changes in rows and thickness of INL in the peripheral retina were smaller than those seen in the central retina, and their prevention by chronic treatment with P021 did not reach significance in 3xTg-21 m/P021 mice (Figures 3B,D,F). In addition, the nuclei of ONL in the central retina were extended across the OPL or protruded into the INL in the WT-21 m/Veh and 3xTg-21 m/Veh mice, which made the gap between the ONL and the INL narrower or unclear (Figures 3Bh,i), whereas this pathology was prevented in 3xTg-21 m/P021 mice (Figure 3Bj). Altogether, these findings indicated the degeneration of both the ONL and the INL in the central and peripheral retinas in WT/Veh and 3xTg/Veh mice at 21 months of age, and the prevention of these changes, especially in the central retina, by chronic treatment with P021 in 3xTg-AD mice.

### P021 Prevents RPE and BM Pathology in Aged Mice

**Figure 4:**
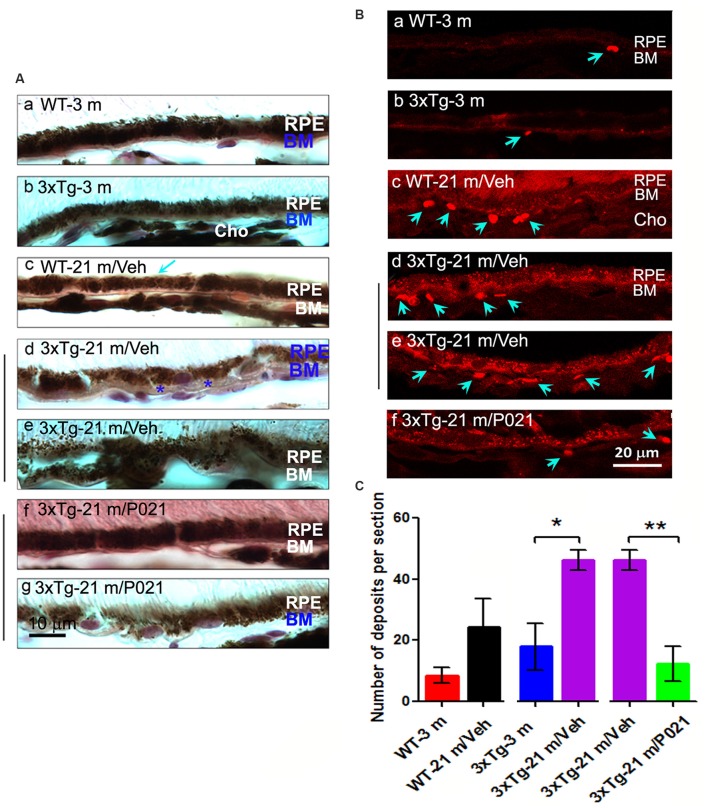
Chronic treatment with P021 prevents AMD-like features of RPE in aged mice. **(A)** Representative H & E image of RPE layer. Thickened (blue asterisk) and atrophic (blue “BM”) BM **(Ae)**. 21-month-old 3xTg/Veh mice showed disarrangement or thinned layer of RPE, full of many brown lipofuscin granules, more than in 21-month-old 3xTg/P021 mice **(Af,g)**, whereas aged WT mice showed a little decrease in the thickness of RPE (**Ac**, blue arrow) as compared with young mice, which showed regular structure and normal arrangement of RPE with no or only a few lipofuscin granules **(Aa,b)**. **(B)** Auto-fluorescence at λ_ex_ = 543 (red channel). Compared with 3-month-old mice and P021-treated 21-month-old 3xTg-mice **(Ba,b,f)**, more deposits (blue arrows) between RPE and Cho, and more lipofuscin granules were detected in 21-month-old WT and 21-month-old 3xTg-mice **(Bc–e)**. **(C)** Quantification of number of deposits in different groups of mice (*n* = 5 mice). Students *t*-test was used to analyze the data between WT-3m and WT-21 m/Veh, between 3xTg-3 m and 3xTg-21 m/Veh, and between 3xTg-21 m/Veh and 3xTg-21m/P021 mice. The data are shown as mean ± SEM. *P < 0.05, **P < 0.01. AMD, age-related macular degeneration; 3xTg, triple-transgenic; RPE, retinal pigment epithelium; BM, Bruch’s membrane; Cho, choroid.

Disruption and degeneration of RPE are well known to occur in AMD (Huang et al., 2017). We, therefore, investigated the occurrence of this pathology in both aged WT and 3xTg-AD mice and the effect of chronic treatment with P021 on its prevention. We found that the areas with the greatest photoreceptor disorganization were often associated with atrophied RPE and increases in lipofuscin granules in the central retina in 3xTg-21 m/Veh mice (Figure 3Bi). The central retina in WT-21 m/Veh and 3xTg-21 m/Veh mice showed multiple additional AMD features, including hypo-pigmentation, thinning, and disorganization of RPE (Figures 3Bi, 4Ad,e). RPE in 3xTg-21 m/Veh mice also showed frequent accumulation of large lipofuscin granules (Figures 3Bi, 4Ae). We found an increase in auto-fluorescence attributable to lipofuscin granules in the aged mice (Figures 4Bc,d,e). We also found thickening of the BM in 3xTg-21 m/Veh mice (Figure 4Ad) compared to young and old WT mice Figures 4Aa–c while there was no BM thickening in 3xTg-21 m/P021 mice (Figures 4Aa,g). Deposits between the RPE and Cho were detected by auto-fluorescence in 3xTg-21 m/Veh mice, much more than in WT-21 m/Veh mice (Figures 4Bd,e); WT-3 m or 3xTg-3 m mice controls, however, showed very few deposits (Figures 4Ba,b). The number of deposits was increased both in WT-21 m/Veh and 3xTg-AD-21 m/Veh mice though in the former it did not reach significance due to large standard error (Figure 4C). The deposits were significantly reduced in 3xTg-21 m/P021 mice, although some small lipofuscin granules could still be observed (Figure 4Bf). Together, our data indicate that aged mice, especially 3xTg-21 m/Veh mice, exhibit RPE and BM abnormalities that highly resemble human dry AMD. Most of these AMD features were prevented by chronic treatment with P021 administered in the diet.

### P021 Reduces Microgliosis in the Retina of Aged Mice and Rats

**Figure 5:**
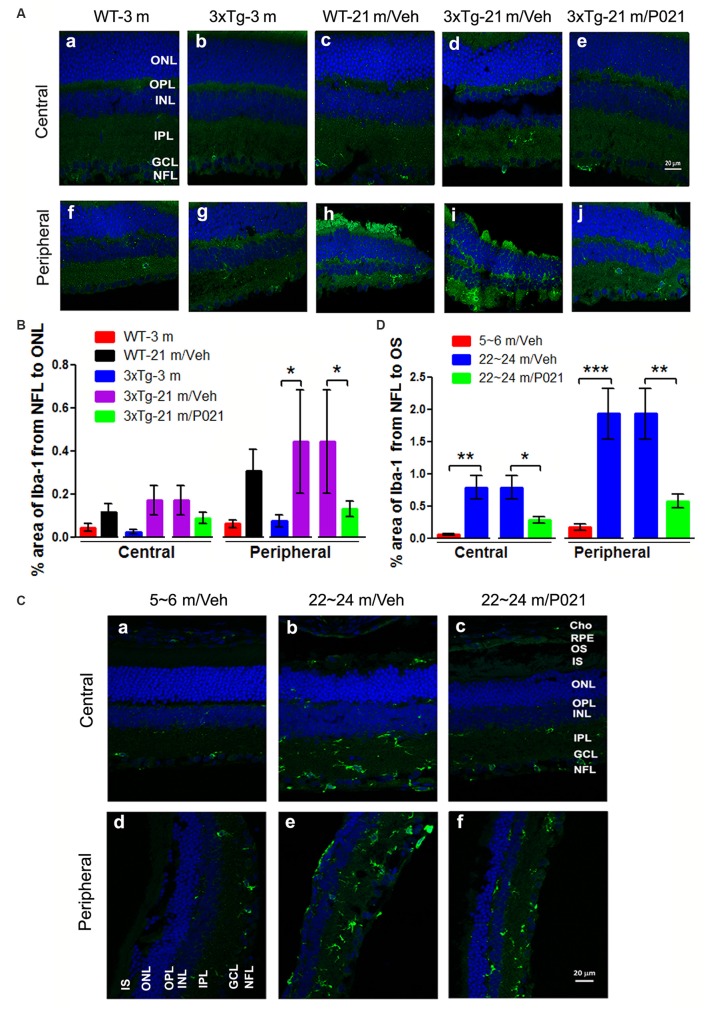
P021 reduces microgliosis in the retinas of aged mice and rats. **(A)** Representative images of Iba-1 immunofluorescence in the central and peripheral retina of mice. Iba-1–positive staining (green) was confined mainly to the NFL and GCL in the retina of 3-month-old mice **(Aa,b,f,g)** and P021-treated 21-month-old mice **(Ae,j)**, whereas it was widely distributed in all the retinal layers from the NFL to the ONL in 21-month-old WT/vehicle and 21-month-old 3xTg/vehicle mice **(Ac,d,h,i)**. **(B)** Quantification of the area percentage of Iba-1 from the NFL to the ONL in the central (*n* = 5–6 mice) and peripheral (*n* = 3–6 mice) retinas of mice. **(C)** Representative images of Iba-1 immunofluorescence in the central and peripheral retinas of rats. Iba-1–positive staining (green) was confined predominately from the NFL to the INL in the retinas of ~5- to 6- month-old/Veh rats **(Ca,d)** and ~22- to 24-month-old/P021 rats **(Cc,f)**, whereas it was widely distributed in all the retinal layers from the NFL to the RPE, and in Cho in ~22- to 24-month-old/Veh rats **(Cb,e)**. **(D)** Quantification of the area percentage of Iba-1 from the NFL to the OS in the central and peripheral retina of rats (*n* = 6–7 rats). The sections were counterstained with TO-PRO 3 iodide, a fluorescent nuclear stain. Student’s *t*-test was used to analyze the data between two groups as shown in **(B,D)**. The data are shown as mean ± SEM. *P < 0.05, **P < 0.01 and ***P < 0.001. WT, wild type; 3xTg, triple-transgenic; AD, Alzheimer’s disease; Cho, choroid; RPE, retinal pigment epithelium; OS, outer segment; IS, inner segment; ONL, outer nuclear layer; OPL, outer plexiform layer; INL, inner nuclear layer; IPL, inner plexiform layer; GCL, ganglion cell layer; NFL, nerve fiber layer.

In the retina, microglial cells form an important part of the immune defense and are quiescent, composed of small and stellate cells strictly limited to the INLs (Lee et al., 2008). In AMD, activated microglia are localized in the ONLs where they are associated with photoreceptor degeneration, apparently for the removal of cell debris (Buschini et al., 2011). We used Iba-1 immunofluorescence to study microgliosis in the retina of aged mice and rats. Iba-1–positive cells were located mainly in the inner layers of the retina (Figure 5). In WT-21 m/Veh mice and 3xTg-21 m/Veh mice, Iba-1–positive staining was distributed into the nerve fiber layer (NFL), ganglion cell layer (GCL), inner plexiform layer (IPL), OPL, and even into the ONL of the central retina (Figures 5Ac,d), whereas in WT-3 m, 3xTg-3 m, and 3xTg-21 m/P021 mice, Iba-1–positive staining was only observed in the GCL; very few Iba-1–positive cells were found in the IPL and other layers (Figures 5Aa,b,e). Moreover, there was an obvious trend of an increase in Iba-1 immunofluorescence from the NFL to the ONL in the central retina in WT-21 m/Veh and 3xTg-21 m/Veh mice (Figure 5B). Although the area percentage of Iba-1–positive staining in the peripheral retina of WT-21 m/Veh, and 3xTg-21 m/Veh was higher than in the young controls (WT-3 m and 3xTg-3 m), these changes showed only a trend towards statistical significance in the WT mice. Interestingly, the Iba-1 immunostaining in 3xTg-21 m/Veh mice was significantly higher than in WT-21 m/Veh mice, whereas it was decreased in 3xTg-21 m/P021 mice to the levels in WT-3 m/Veh and 3xTg-3 m/Veh mice in the peripheral retina (Figure 5B), even though there was no obvious difference in the distribution of Iba-1–positive staining (Figures 5Af–j).

We found Iba-1–positive microglial cells not only in the whole retinal layers, including the NFL, IPL, INL, OPL, ONL, and even the IS/outer segment (OS), but also in the Cho of the ~22- to 24-month-old/Veh rats (Figures 5Cb,e), but the 5–6 m/Veh rats showed a limited location of Iba-1 only from the NFL to the INL, and the Cho (Figures 5Ca,d). In contrast, ~22- to 24-month-old/P021 rats showed a decrease in localization from the NFL to the OPL compared with ~22- to 24-month-old/Veh rats, and no staining was found from the ONL to the OS, especially in the central retina after P021 treatment (Figures 5Cc,f). Quantitatively, the relative Iba-1 immunostaining from the NFL to the OS in the central and peripheral retinas of ~22- to 24-month-old/Veh rats was increased significantly in ~22- to 24-month-old/Veh rats as compared to ~5- to 6-month-old/Veh rats, and P021 treatment dramatically reduced it in ~22- to 24-month-old rats (Figure 5D).

Altogether, these findings indicate that microglia are distributed more widely in the whole retina of aged mice and rats, especially in the ONL, which is associated with photoreceptor degeneration (shown in Figures 1, 3), and that treatment with P021 can efficiently protect the retina from inflammatory damage in aged mice and rats.

### P021 Reduces Astrogliosis in the Retina of Aged Mice and Rats

**Figure 6:**
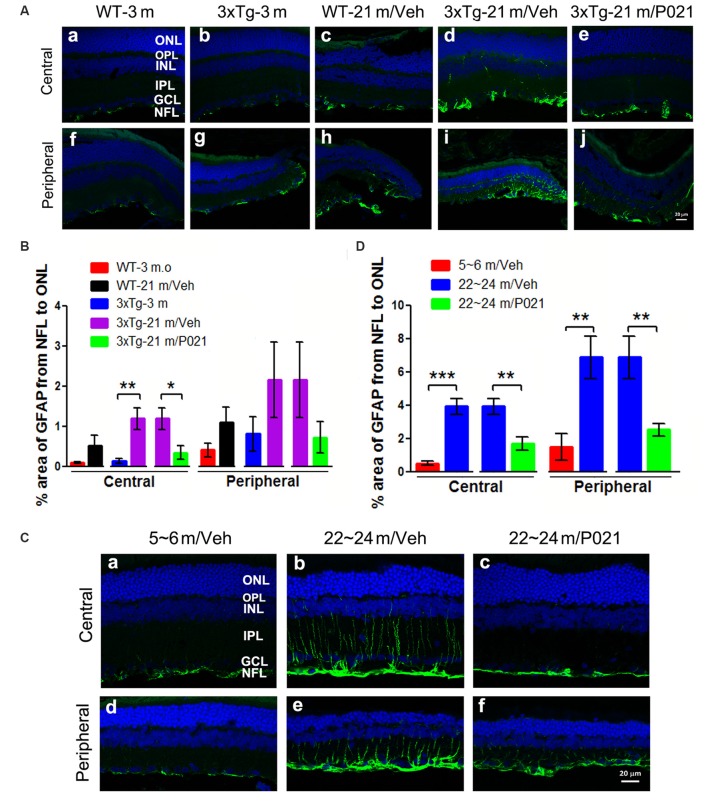
P021 reduces astrogliosis in the retinas of aged mice and rats. **(A)** Representative images of GFAP immunofluorescence in the central and peripheral retina of mice. GFAP-positive staining (green) was confined predominately to the NFL and GCL in the retina of 3-month-old/vehicle WT and 3xTg mice **(Aa,b,f,g)**, whereas it was widely distributed from the NFL to the OPL in 21-month-old WT/Veh mainly peripheral **(Ac)** and 21-month-old 3xTg/Veh mice **(Ad,h,i)** and was reduced in 21-month-old 3xTg/P021 mice **(Ae,j)**. **(B)** Quantification of the area percentage of GFAP from the NFL to the ONL in the central (*n* = 5–6 mice) and peripheral (*n* = 3–6 mice) retinas of mice. **(C)** Representative images of GFAP immunofluorescence in the central and peripheral retinas of rats. GFAP-positive staining (green) was confined mainly from the NFL to the INL in the retinas of ~5- to 6-month-old/Veh rats **(Ca,d)** and ~22- to 24-month-old/P021 rats **(Cc,f)**, whereas it was widely distributed from the NFL to the OPL in ~22- to 24-month-old/Veh rats **(Cb,e)**. **(D)** Quantification of the area percentage of GFAP from the NFL to the OS in the central and peripheral retina of rats (*n* = 6–7 rats). The sections were counterstained with TO-PRO 3 iodide, a fluorescent nuclear stain. Student’s *t*-test was used to analyze the data between two groups as shown in **(B,D)**. The data are shown as mean ± SEM. *P < 0.05, **P < 0.01 and ***P < 0.001. WT, wild type; 3xTg, triple-transgenic; GFAP, glial fibrillary acidic protein; Cho, choroid; RPE, retinal pigment epithelium; OS, outer segment; IS, inner segment; ONL, outer nuclear layer; OPL, outer plexiform layer; INL, inner nuclear layer; IPL, inner plexiform layer; GCL, ganglion cell layer, NFL, nerve fiber layer.

Astrocytes are another key cell type involved in macular degeneration. Previous studies showed activated astrocytes in the retina of Tg2576 mice at 14 months (Liu et al., 2009) and of 3xTg-AD mice at 9 months and ~18–24 months (Edwards et al., 2014). To study the localization of astrogliosis and the response to P021 treatment in the retina of aged mice and rats, astrocytic/Müller cell activation was evaluated by immunostaining with anti-glial fibrillary acidic protein (GFAP). GFAP-positive staining was confined to astrocytes in the GCL of young mice (Figures 6Aa,b,f,g) and WT-21 m/Veh mice (Figures 6Ac,h) but was highly elevated in Müller glial cells in the INL and other layers such as the IPL, OPL, and ONL of the central and peripheral retina in 3xTg-21 m/Veh mice (Figures 6Ad,i). In contrast, the GFAP-positive staining was limited to the NFL and GCL in 3xTg-21 m/P021 mice (Figures 6Ae,j). The area percentage of GFAP from the NFL to the ONL in the central retina of 3xTg-21 m/Veh mice was significantly higher than in 3xTg-3 m mice and WT-21 m/Veh mice and was reduced by P021 treatment to the levels in WT-3 m/Veh and 3xTg-3 m/Veh mice (Figure 6B). The central astrocytosis was also increased in WT-21 m/Veh as compared to WT-3 m/Veh, but the difference did not reach statistical significance. There was also a similar trend in the peripheral retina between groups, but the changes did not reach statistical significance due to large standard error (Figure 6B).

We also studied retinal astrogliosis by GFAP immunostaining in rats. We found that the area of GFAP-positive staining was extended from the NFL to the ONL in the central and peripheral retinas of ~22- to 24-month-old/Veh rats (Figures 6Cb,e), but the staining was considerably less and was limited to the NFL and GCL in ~5- to 6-month-old/Veh rats (Figures 6Ca,d). The GFAP staining was reduced very much in ~22- to 24-month-old /P021 rats as compared to ~22- to 24-month-old/Veh rats (Figures 6Cc,f). The area percentage of GFAP-positive staining was also drastically elevated in the central and peripheral retinas in ~22- to 24-month-old/Veh rats as compared to ~5- to 6-month-old/Veh rats and ~22- to 24-month-old/P021 rats (Figure 6D).

Altogether, the above findings indicate that P021 can prevent and rescue microgliosis and astrogliosis in the retina of both aged 3xTg-mice and aged rats.

### P021 Affects Alzheimer-Like Pathology and VEGF Changes in the Retina and Optic Nerve of Aged Rats and Mice

Tau and Aβ pathologies are well-known hallmarks of AD. Previous studies showed both tau and Aβ pathologies in the retina of 14 m Tg2576 mice (Liu et al., 2009; Hart et al., 2016) and APP immunoreactivity in the retina in a number of AD animal models, including Tg mouse models (hTgAPP^tg/tg^, APP_SWE_/PS1_ΔE9_, and APP_SWE_/PS1_M146L/L286V_) that exhibit several features of AD (Frederikse and Ren, 2002; Ning et al., 2008; Dutescu et al., 2009; Zhao et al., 2013; Du et al., 2015). Moreover, several studies found Aβ deposition in the drusen or retinal layers in AMD animal models (Crabb et al., 2002; Prasad et al., 2017). Anti-Aβ immunotherapy was reported to reduce ocular Aβ deposits in a mouse model of AMD (Ding et al., 2011). In the present study, we investigated the presence and the effect of P021 treatment on AD pathology in the retina. We found tau and Aβ pathologies in the retinas of aged rats and 3xTg-mice: immunofluorescence results showed that after normalization with negative controls (Supplementary Figure S1), positive staining of total tau detected by rabbit polyclonal antibody R134d (Supplementary Figure S2) and hyperphosphorylation of tau at Ser-396/404 by mouse monoclonal antibody PHF-1 (Supplementary Figure S3) were increased in the sub-retinal layers of aged rats and 3xTg-mice, and even 3xTg-21 m/Veh showed increased PHF-1 immunoreactivity in the optic nerve (Supplementary Figure S3B, lower panel). Hyperphosphorylation of tau at Ser-202/Thr-205, detected with mouse monoclonal antibody AT8 (Supplementary Figure S4), and of Aβ/APP, detected by mouse monoclonal antibody 4G8 and rabbit monoclonal Aβ (Supplementary Figure S5), was also highly expressed in sub-retinal layers. The positive staining was not sharp and condensed, which appeared as small particles, especially PHF-1, AT8, 4G8, and Aβ immunostaining in the retina and optic nerve in mice, but in comparison with the negative controls (Supplementary Figures S1C,D), the positive staining was specific. Significantly, P021 treatment prevented the trend in the increase in tau and Aβ pathologies in the aged mice and rats (Supplementary Figures S2–S5).

Late-stage dry AMD and wet AMD can coexist in AMD development (Kaszubski et al., 2016). Excessive amounts of vascular endothelial growth factor (VEGF) is a major pathological change in wet AMD, and anti-VEGF can relieve or delay the progress of wet AMD (Xu et al., 2015; Wang et al., 2016). Anti-VEGF treatment of the combined dry/wet AMD phenotype was reported to be effective in a case series of 11 eyes (Amaro and Roller, 2012). In the present study, we found that VEGF-positive spots were located mainly in the inner retinal layers from the NFL to the IPL and in the RPE in young mice, but they were more widely distributed in the INL, OPL, and ONL in aged mice and less so in the RPE in aged WT-21 m/Veh and 3xTg-21 m/Veh mice (Supplementary Figure S6). In 3xTg-21 m/P021 mice, the localization of VEGF-positive spots was limited mainly to the GCL to the IPL, similar to in the young control animals. In the optic nerve, aged 3xTg-21 m/Veh mice showed an increase in VEGF staining, and the size of the staining patch was decreased after P021 treatment. While tau and Aβ pathologies found in 3xTg-AD-21 m/Veh and VEGF changes in both WT-21 m/Veh and 3xTg-AD-21 m/Veh mice were consistent with the previous literature and appeared specific, they displayed wide scatter and were therefore not quantified and pursued beyond 2–3 animals/group.

## Discussion

In this study, we found for the first time several AMD-like features that recapitulate human dry AMD, including photoreceptor cell loss (decrease in rows and thickness of ONL), rosette-like formation in the photoreceptor cell layer, RPE disruption, accumulation of lipofuscin and vacuoles in RPE, increase in auto-fluorescence of RPE, BM thickening, and the formation of basal deposits between RPE/BM and Cho in aged rats, mice and 3xTg-AD mice. P021 conferred protection for the retina against this age- and disease-related damage. Even neuroinflammation detected by microgliosis and astrocytosis was ameliorated by P021 treatment. Furthermore, P021 prevented the increase in tau and Aβ pathologies and VEGF deposition in the sub-retina. These findings are the first demonstration of AMD-like pathology in aged rats, and 3xTg-AD mice and its rescue by a neuro-regenerative compound, P021.

Several studies reported a decrease in rows and thickness of the ONL or retinal thickness in different animal models of AMD. The thickness of the ONL in 24-month-old mice treated with a high glycemic diet was decreased (Rowan et al., 2017). Rows of ONL were reduced in 4-month-old C57BL/6 mice after intraocular injection of Aβ40/42 (Prasad et al., 2017). Another group found that retinal thickness was significantly decreased in 14-month-old Tg2576 mice (Liu et al., 2009). The decreased number of rows and reduced thickness in the ONL seen by H & E staining in aged rats and mice in the present study are in agreement with these findings. Moreover, the present study also showed, by Iba-1 and GFAP immunofluorescence counterstained with To-Pro, a decrease in the rows and thickness of the ONL and INL in aged mice and rats, and P021 treatment prevented these changes. These degenerative changes in the ONL and INL might occur only in the aged animals, because we did not find these changes in 3-month-old mice or in 5- to 6-month-old rats, and a previous study reported no significant changes in rows and thickness of the retinal layer in APP_SWE_/PS1_ΔE9_ mice at ~9–12 months of age (Chidlow et al., 2017).

RPE pathology is one of the pivotal AMD features, and drusen or drusen-like deposition between RPE and BM was reported in AMD in humans and some animal models (Anderson et al., 2002; Luibl et al., 2006; Markovets et al., 2011; Ardeljan and Chan, 2013; Huang et al., 2017). In the present study, we did not find the drusen-like pathology in aged 3xTg-mice and in aged rats, possibly because of either the small sample size or the animal models used. In fact, not all models of AMD exhibit the typical morphology, and drusen is not uniquely associated with AMD (Pennesi et al., 2012). Interestingly, we found a rosette-like formation in ~22- to 24-month-old rats, similar to the previous findings that were reported in the retina of a 92-year-old male with AMD (Rayborn et al., 1997) and in a 3-month-old Rdh8^−/−^/Abca4^−/−^ mouse retina stained with H & E (Flynn et al., 2014). Our finding of rosette-like structures is consistent with the disorganized and fragmented ONL or photoreceptors reported in 24-month-old OXYS rats (Markovets et al., 2011).

In addition, we also observed BM thickening, atrophy, and vacuolization of RPE in 21-month-old 3xTg/Veh-mice and ~18- to 24-month-old rats, which is consistent with previous studies in 17-month-old Cxcr5^−/−^ mice and a 27-month-old neprilysin-deficient mouse model of AMD (Yoshida et al., 2005; Huang et al., 2017). Senescence-accelerated OXYS rats had atrophic areas in the RPE at 1.5 months of age, and some animals developed thickened BM by 12 months and obvious atrophy at 24 months (Neroev et al., 2008; Markovets et al., 2011). However, it is important to note that RPE vacuolization in human AMD is actually quite rare and is not considered a phenotypic feature of the disease (Zanzottera et al., 2015), although it has been reported in mouse models of AMD.

In spite of some studies that reported that lipofuscin deposits develop with aging (Delori, 1998; Julien and Schraermeyer, 2012), several reports showed that lipofuscin accumulation in the RPE is an important change in individuals with AMD and in mouse models (Dorey et al., 1989; Rowan et al., 2017). Increased lipofuscin granules in RPE cells were also observed in 11- to 13-month-old OXYS rats and in 24-month-old Wistar rats (Markovets et al., 2011). In the present study, we found that not only H & E staining but also auto-fluorescence exhibited a drastic increase in lipofuscin particles in the RPE and a large number of deposits between the RPE/BM and Cho in aged rats and 3xTg-mice. These findings suggest that preferential accumulation of lipofuscin in the RPE or the deposits outside the RPE may be due to, to a limited extent, the increased phagocytic and metabolic load on the RPE, which ultimately leads to photoreceptor cell loss or degeneration of other cellular layers, as was reported previously (Dorey et al., 1989).

Clinically, fundus auto-fluorescence has proven to be valuable in the diagnosis and differentiation of retinal disease (Sparrow and Duncker, 2014). Post-mortem AMD retinas showed increased autofluorescence in the RPE, BM, or Cho structures, and the excitation fluorescence was more at 510 nm than at 470 nm (Anderson et al., 2002; Marmorstein et al., 2002). In the present study, we found the formation of thickened BM detected by H & E and auto-fluorescence detected at λ_ex_ = 542 nm, and increased auto-fluorescence in the OS and IS in aged mice. The RPE lipofuscin is known to be produced in the membranes of outer segments (Frost et al., 2016) from the non-enzymatic reactions of vitamin A aldehyde (Katz et al., 1987; Eldred and Katz, 1988; Ben-Shabat et al., 2002). This fluorescent material is transferred to RPE cells within phagocytosed OS disks (Young and Bok, 1969; Katz et al., 1986) and becomes deposited in the lysosomal compartment of the cells or secreted to the outside of the RPE. These granules are potentially phototoxic and cause further RPE degeneration (Markovets et al., 2011).

AD pathology includes astrogliosis and microglial activation (Rodríguez et al., 2009; Heneka et al., 2010; Verkhratsky et al., 2010). In contrast, in the normal adult retina, microglia are quiescent, composed of small and stellate cells limited to the inner retinal layers (Lee et al., 2008). The present study showed that in the retinas of WT-21 m and 3xTg-21 m mice and ~18- to 24-month-old rats, the distribution and intensity of microglial cells in the sub-retinal layers were higher than those in the young controls. The increased microgliosis and wide distribution in aged mice and rats are partially in agreement with findings in the previous studies in different AMD models (Chen and Xu, 2015; Prasad et al., 2017).

Impairment of microglial migration into or out of the sub-retinal space is known to promote the death of photoreceptor cells (Tuo et al., 2007; Chen et al., 2011). In AMD, microglia accumulate in the subretinal space (Xu et al., 2009); this is probably a symptom of inflammatory damage that exacerbates retinal degeneration (Ambati et al., 2013).

Another key glial cell type in the retina is astrocytes, and we found that the distribution and intensity of GFAP were significantly elevated in the whole retinal layers and displayed a hypertrophic and reactive morphology in aged mice and rats. These findings support the earlier report of increased GFAP-positive cell processes in the retinas of 3xTg-AD mice ~18–24 months of age compared to those seen at 9 months (Edwards et al., 2014). The data presented here suggest that Müller cells and astrocytes in the retina undergo complex remodeling similar to astrocyte changes in the brain in AD. Further studies are required to fully understand the consequences of glial activation in 3xTg-AD mouse retina and to determine how these changes correlate with tangles or Aβ amyloid deposition.

Numerous studies examining the retinas of sporadic and transgenic animal models of AD have reported Aβ deposits and hyperphosphorylated tau, often in association with retinal ganglion cell degeneration, local inflammation (i.e., microglial activation), impairments of retinal structure and function (Alexandrov et al., 2011; Koronyo-Hamaoui et al., 2011; Zhao et al., 2013; Tsai et al., 2014; Du et al., 2015; Pogue et al., 2015; Hart et al., 2016), and an increase in cytoplasmic AβPP in the photoreceptor layer in transgenic rodents (Ning et al., 2008; Dutescu et al., 2009). These studies, which included a variety of transgenic rat and mouse models, demonstrated abundant tau and Aβ deposits mainly in the innermost retinal layers (GCL and NFL; Tsai et al., 2014; Du et al., 2015; Gupta et al., 2016). In the present study, we found that there was a consistent trend in the increase in total tau, hyperphosphorylated tau, and Aβ immunostaining in the retina in aged rats and 3xTg-AD mice, and P021 treatment seemed to alleviate these pathological changes. These findings are consistent with our previous studies which showed that P021 improves AD-like pathology in the brains of AD rat and mouse models (Kazim et al., 2014; Khatoon et al., 2015). Although in the current study we found hyperphosphorylated tau and Aβ immunofluorescence in the retinas of aged rats and 3xTg-mice, both of them were weak and patchy; these findings are consistent with previous reports (Koronyo-Hamaoui et al., 2011; Chiu et al., 2012; Ho et al., 2012; Edwards et al., 2014). In a previous study, tau was detected in the sub-retinal layers from the NFL to the OPL, even in the RPE of young and old humans, but the immunoreactivity was weak or patchy (Löffler et al., 1995). Furthermore, it was suggested that Aβ and tau pathologies, combined with gliosis, drive neurodegeneration in AD (Leyns and Holtzman, 2017).

While in the present study we found Aβ immunostaining with two monoclonal antibodies, one mouse and another rabbit IgG and our findings are consistent with several similar studies, biochemical studies on the affected tissue in the future will be required to confirm these changes; mouse monoclonal Aβ antibody 4G8 used in the present study has been reported to immunostain besides Aβ and APP; fibrils formed from alpha-synuclein and Islet Amyloid Polypeptide and cross-reactivity of other Aβ monoclonals have been suggested (Hatami et al., 2016); (Hunter and Brayne, 2017).

In the present study, we also detected an increase in VEGF immunostaining in the retinas of aged mice, which is consistent with previous studies that reported that anti-VEGF therapy is the standard of care for symptomatic wet AMD and can significantly improve visual acuity (Avery et al., 2006; Rosenfeld et al., 2006). Macrophages such as microglia, together with RPE cells, are a major source of pro-angiogenic factors, such as VEGF A, and the production of VEGF (Shen et al., 1998). In the case of the breakdown of the blood-retina barrier, macrophage cells modulate the disease through their recruitment from the underlying Cho or from the systemic circulation into the retina (Ambati et al., 2013). Thus, the increased microgliosis and astrogliosis found in the present study could have promoted the secretion and migration of VEGF into the subretinal layers and exacerbated the progression of dry AMD and led to the combined phenotype of dry/wet AMD.

Most of the pathological changes in morphology described above were prevented or reversed by P021 treatment, although some remained in the central retina and others in the peripheral retina. Also, microgliosis and astrogliosis in the sub-retina of aged rats and 3xTg-AD mice were ameliorated by P021 treatment. In addition, VEGF pathology showed an obvious trend of reduction by P021 treatment. Neuroinflammation in 3xTg-AD mice has also been shown to correspond to ONL degeneration (Tuo et al., 2007; Chen et al., 2011). Aβ or tau pathology may be the stimulator of neuroinflammation (Bronzuoli et al., 2016; Ardura-Fabregat et al., 2017). Moreover, the RPE is pivotal for maintaining the structure and function of the retina. The early alteration of RPE cells can be a key factor for the development of the retinopathy in aged rats and mice and a cause of all subsequent pathological changes. Therefore, abnormalities of the retina could be initially caused by the enhanced neuroinflammatory response, including infiltration of inflammatory cells and local edema, which leads to the RPE disruption and then the pathology in the sub-retinal layers. It is thus possible that P021, by virtue of its effect on retinal pathologies, normalized the tau/Aβ–induced neuroinflammation in aged 3xTg-AD mice and rats, which in turn contributed to the beneficial effect of P021 on the pathological changes of the RPE, neuronal loss in the retina, especially in the ONL and INL, and even the whole retina.

The PI3K-Akt-GSK-3 pathway was reported to be insensitive in peripheral blood mononuclear cells of AMD patients and in cultured RPE cells (Busch et al., 2017; Liu and Yao, 2017; Zheng et al., 2018). In our previous study, we found that P021 inhibited the tau and Aβ pathologies by increasing BDNF-mediated activation of TrkB-PI3K-Akt-GSK-3β signaling (Kazim and Iqbal, 2016). Thus, in the present study, oral administration of P021 could have increased the activity of PI3K-Akt-GSK-3 signaling in the blood circulation system or in the RPE of the aged rats and mice, and thereby prevented the AMD-like pathology.

In the present study, aged rats and 3xTg-AD mice were found to develop multiple pathological features of dry AMD from the aspect of morphology and immunohistochemistry. However, there are a few inherent limitations of this study. First, rodents do not have a macula/fovea and thus cannot completely mimic the human AMD condition. Second, no visual ability or electroretinography and biochemical analysis were conducted. Some of the retinal changes we found could be associated with aging and AD. Although our qualitative observations suggest that P021 may ameliorate tau, Aβ amyloid and VEGF components of retinal pathology, in-depth and further biochemical analysis in the future will be needed to confirm and extend these findings. Finally, some effects of P021 were rather small in magnitude; they were perhaps related to the amount of damage in AMD and/or the dose of P021. Nevertheless, our study suggests that chronic treatment with P021 is an effective and attainable way to prevent or inhibit AMD-like pathology and retinal changes associated with aging and AD. Moreover, our findings demonstrate aged rats and mice as new models of AMD that can be employed for preclinical studies to test compounds for therapeutic intervention of AMD.

## Ethics Statement

All procedures involving rats and mice were reviewed and approved by the Institute for Basic Research in Developmental Disabilities Animal Care and Use Committee (protocol number 198) and were carried out according to the guidelines of the National Institutes of Health.

## Author Contributions

YL carried out the study and wrote the article. WW studied the autofluorescence and analyzed the data. NB treated the animals and extracted the eyeballs and fixed them in paraformaldehyde. FL helped in discussing the data and writing the article. KI conceived, designed, supervised the study and wrote the article.

## Disclosure

The authors have a U.S. patent on neurotrophic peptides, which includes compound P021, and additional patent-related applications are in the process. The animal studies conform to the National Institutes of Health guidelines and were approved by our institutional IACUC.

## Dedication

This paper is dedicated to Dr. Inge Grundke-Iqbal, who along with KI conceived this study. Dr. Grundke-Iqbal passed away on September 22, 2012.

## Conflict of Interest

The authors declare that the research was conducted in the absence of any commercial or financial relationships that could be construed as a potential conflict of interest.

## Footnotes

^1^https://www.jax.org/strain/004807
