# Supplementary material for: Inhibition of AMD-Like Pathology With a Neurotrophic Compound in Aged Rats and 3xTg-AD Mice
Source: Front Aging Neurosci. 2019 Nov 19;11:309. doi: 10.3389/fnagi.2019.00309 (PMC6877482; doi:10.3389/fnagi.2019.00309)
Supplement: Supplementary file 1 [file Table_1.DOC]

**Inhibition of AMD-like pathology with a neurotrophic compound in aged rats and**

**3xTg-AD mice**

**Supplementary figure legends**

**Fig. S1. Negative controls of immunofluorescence without primary antibodies. A, B.** Representative images of immunofluorescence of retinas of ~ 22- to 24-month-old/Veh rats in the absence of any primary antibodies by using goat anti-rabbit (GAR) or goat anti-mouse (GAM) secondary antibodies. **C, D.** Representative images of immunofluorescence in the retinas of 3xTg-21m/Veh mice by using GAR or GAM secondary antibody. **E.** Representative image of immunofluorescence in the optic nerves of 3xTg-21m/Veh mice by using GAM secondary antibody. The sections were counterstained with TO-PRO 3 iodide, a fluorescent nuclear stain. Not shown in this figure, no primary antibodies control immunostaining was conducted on all five groups of mice were performed, and like the representative images shown in this figure only background was observed. GAR, Alexa Fluor 488-conjugated goat anti-rabbit IgG; GAM, Alexa Fluor 488-conjugated goat anti-mouse IgG; 3×Tg, triple-transgenic; IS, inner segment; ONL, outer nuclear layer; OPL, outer plexiform layer; INL, inner nuclear layer; IPL, inner plexiform layer; GCL, ganglion cell layer; NFL, nerve fiber layer.

**Fig. S2. P021 prevents the increase in total tau immunoreactivity in retinas of aged rats and mice. A.** Representative images of immunofluorescence (green) with rabbit polyclonal tau antibody R134d in the central (upper panels) and peripheral retinas (lower panels) of rats. **B.** Representative images of R134d immunofluorescence in the central (upper panels) and peripheral retinas (lower panels) of mice. The sections were counterstained with TO-PRO 3 iodide, a fluorescent nuclear stain. WT, wild type; 3×Tg, triple-transgenic; m, month; Veh, vehicle; ONL, outer nuclear layer; OPL, outer plexiform layer; INL, inner nuclear layer; IPL, inner plexiform layer; GCL, ganglion cell layer; NFL, nerve fiber layer.

**Fig. S3. P021 prevents the increase in the accumulation of tau hyperphosphorylation at Ser-396/404 (PHF-1site) in retinas of aged rats and mice. A.** Representative images of PHF-1 immunofluorescence (green) in the central (upper panels) and peripheral retina (lower panels) of rats. **B.** Representative images of PHF-1 immunofluorescence (green) in the central (upper panels) and optic nerve (lower panels) of mice. The sections were counterstained with TO-PRO 3 iodide, a fluorescent nuclear stain. WT, wild type; 3×Tg, triple-transgenic; m, month; Veh, vehicle; ONL, outer nuclear layer; OPL, outer plexiform layer; INL, inner nuclear layer; IPL, inner plexiform layer; GCL, ganglion cell layer; NFL, nerve fiber layer.

**Fig. S4. P021 prevents the increase in the accumulation of tau hyperphosphorylation at Ser-202/Thr-205 (AT8 site) in retinas of aged rats and mice. A.** Representative images of AT8 immunofluorescence in the central retinas of rats. **B.** Representative images of AT8 immunofluorescence in the central and peripheral retinas (from same eye) of mice. **C.** Representative images of AT8 immunofluorescence in the optic nerve of 3xTg-mice. The sections were counterstained with TO-PRO 3 iodide, a fluorescent nuclear stain. WT, wild type; 3×Tg, triple-transgenic; m, month; Veh, vehicle; RPE, retinal pigment epithelium; OS, outer segment; IS, inner segment; ONL, outer nuclear layer; OPL, outer plexiform layer; INL, inner nuclear layer; IPL, inner plexiform layer; GCL, ganglion cell layer; NFL, nerve fiber layer.

**Fig. S5. P021 prevents the increase of Aβ/APP immunoreactivity in retinas of aged rats and mice. A**. Representative images of immunofluorescence with anti Aβ (4G8) in the central retinas of rats. **B.** Representative images of 4G8 immunofluorescence in the central retinas of mice. **C.** Representative images of Aβ immunofluorescence with a rabbit monoclonal to Aβ1-42 in the central (upper panel) and peripheral (lower panel) retinas of mice. The sections were counterstained with TO-PRO 3 iodide, a fluorescent nuclear stain. m, month; Veh, vehicle; ONL, outer nuclear layer; OPL, outer plexiform layer; INL, inner nuclear layer; IPL, inner plexiform layer; GCL, ganglion cell layer; NFL, nerve fiber layer.

**Fig. S6. P021 prevents the increase in the expression of VEGF in retinas of aged mice.** Representative images of **VEGF** immunofluorescence in central retinas (upper panel) and optic nerves of rats (lower panel). VEGF-positive particles were observed from GCL to IPL in different groups; OS and IS show strong autofluorescence. The sections were counterstained with TO-PRO 3 iodide, a fluorescent nuclear stain. WT, wild type; 3×Tg, triple-transgenic; m, month; Veh, vehicle; RPE, retinal pigment epithelium; OS, outer segment; IS, inner segment; ONL, outer nuclear layer; OPL, outer plexiform layer; INL, inner nuclear layer; IPL, inner plexiform layer; GCL, ganglion cell layer; NFL, nerve fiber layer.


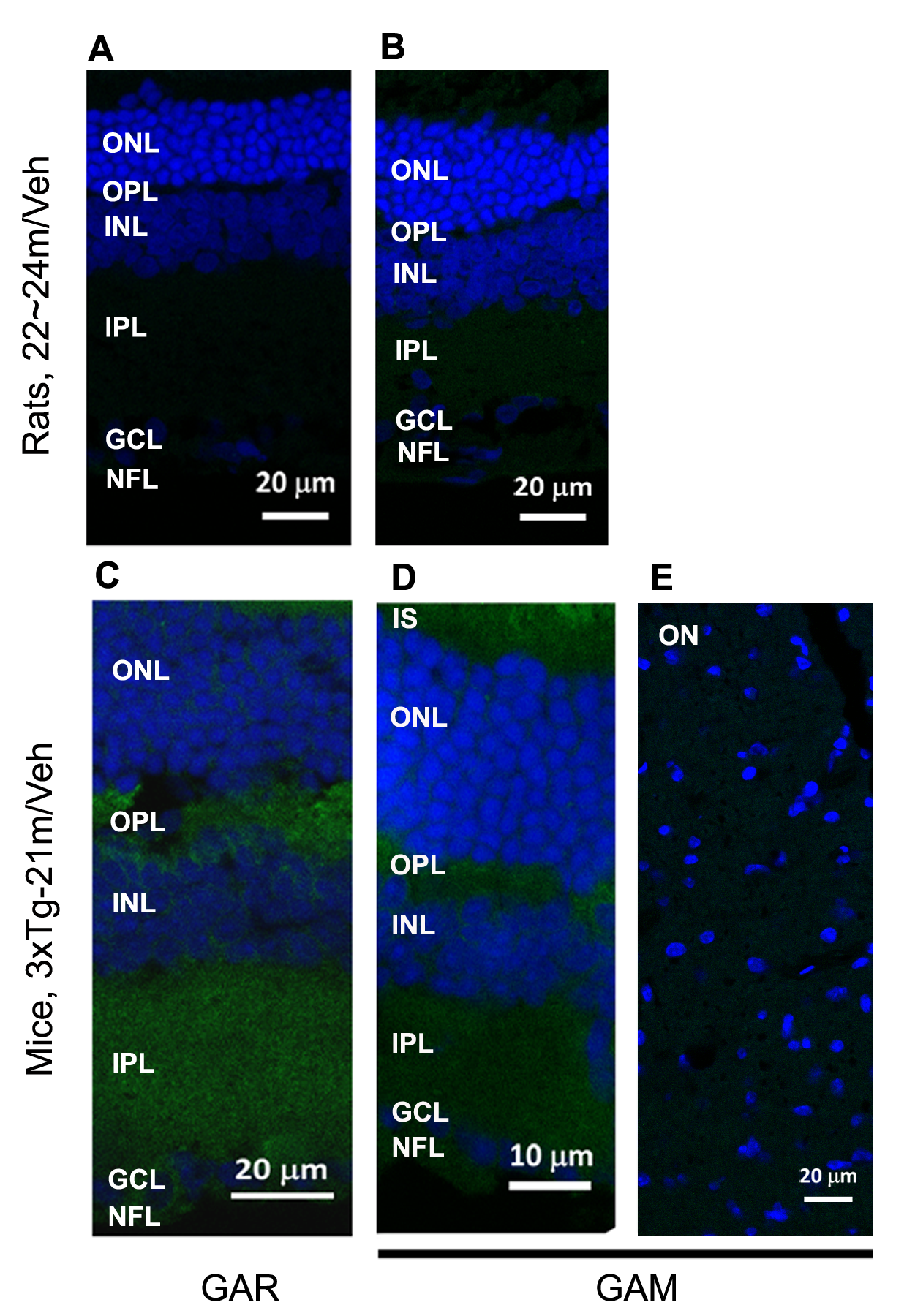


Fig. S1


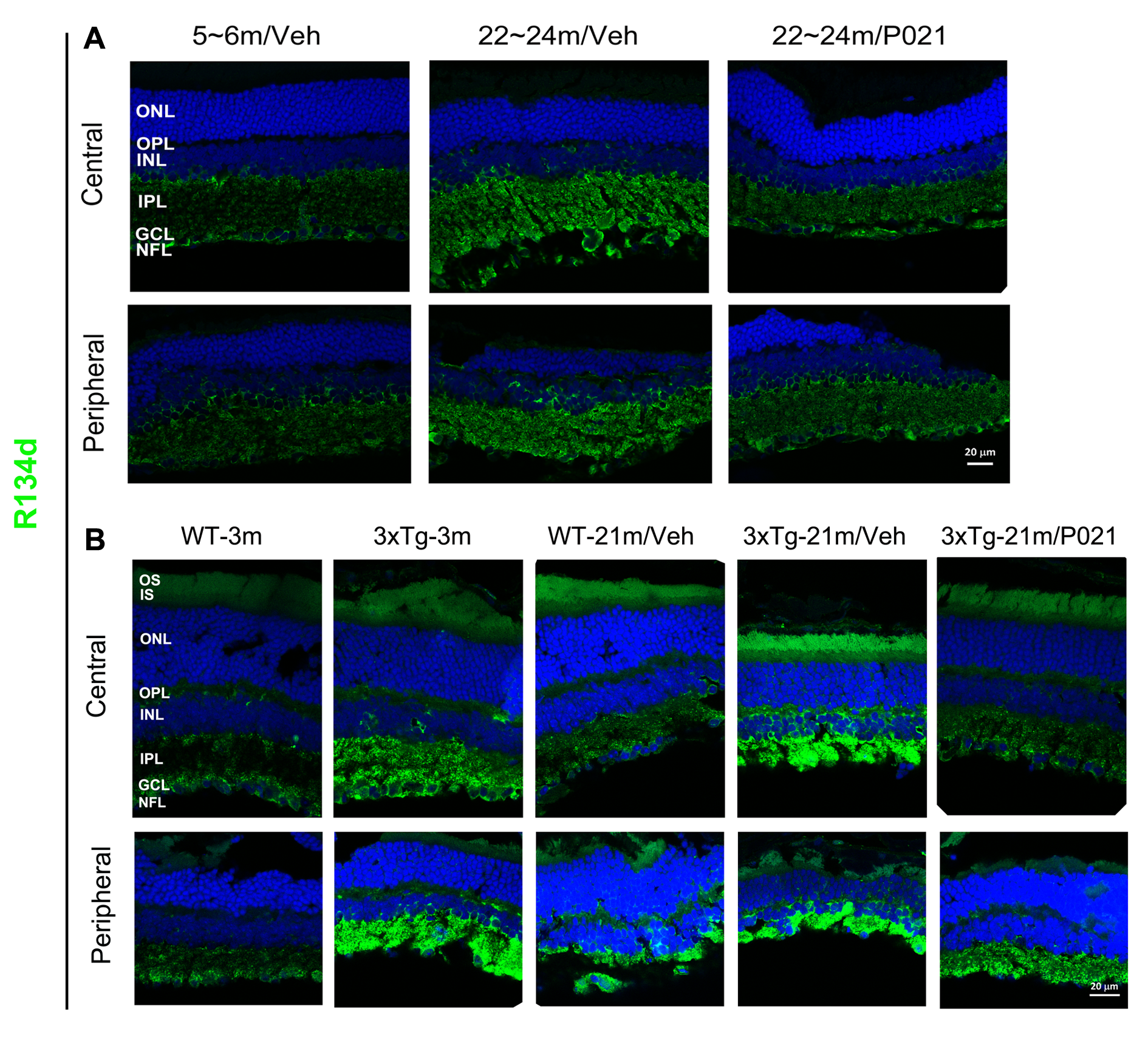


Fig. S2


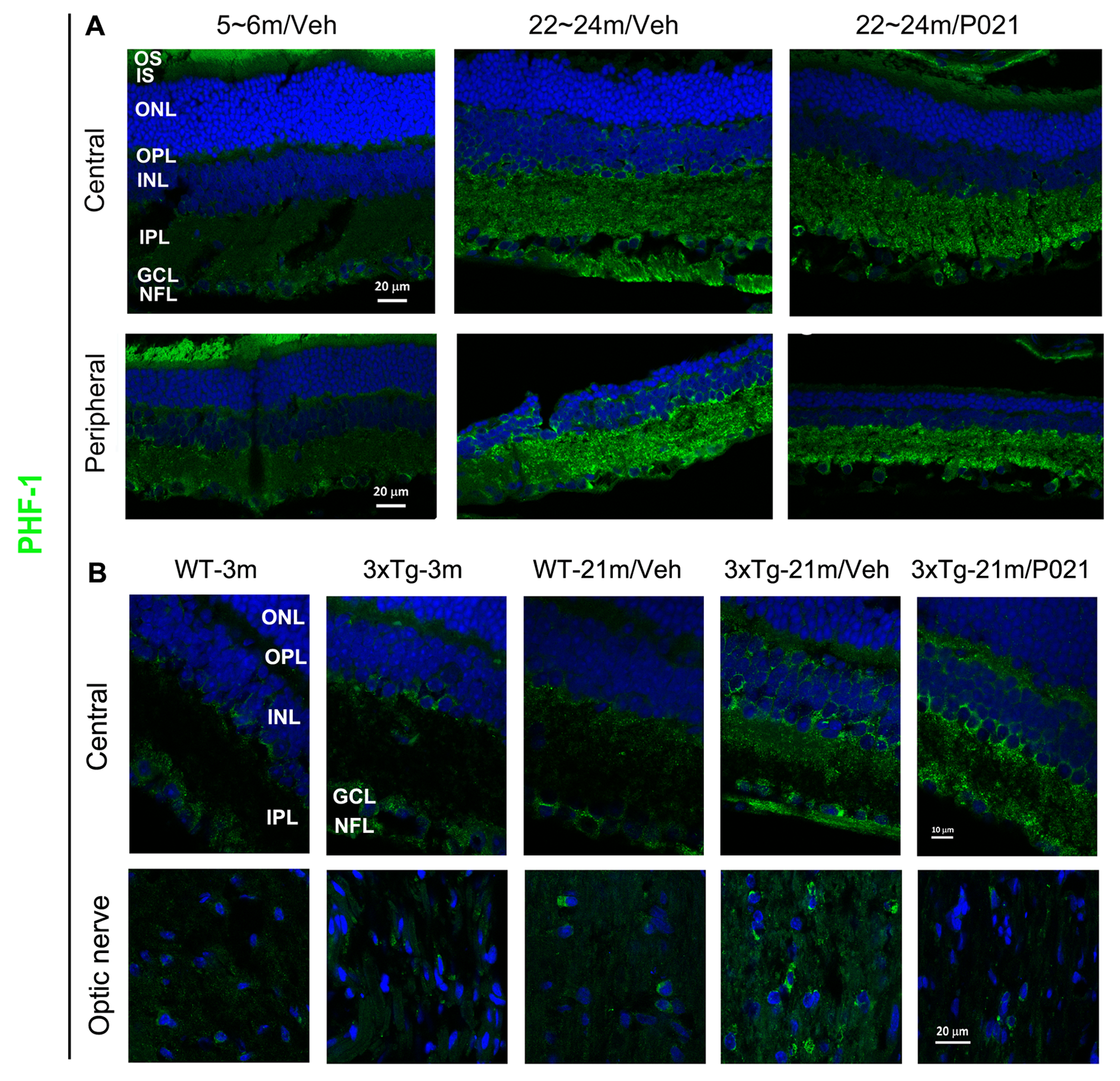


Fig. S3


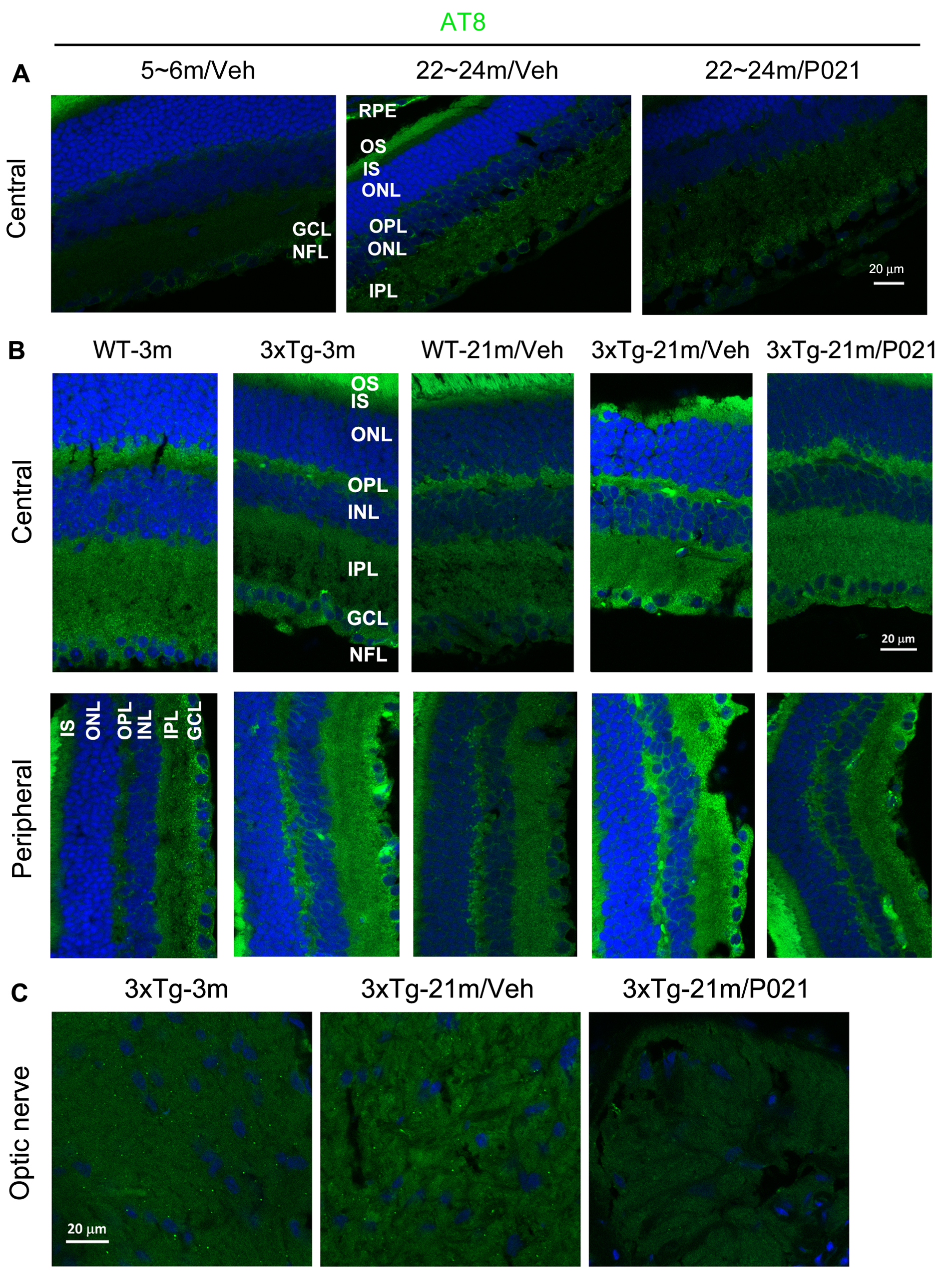


Fig. S4


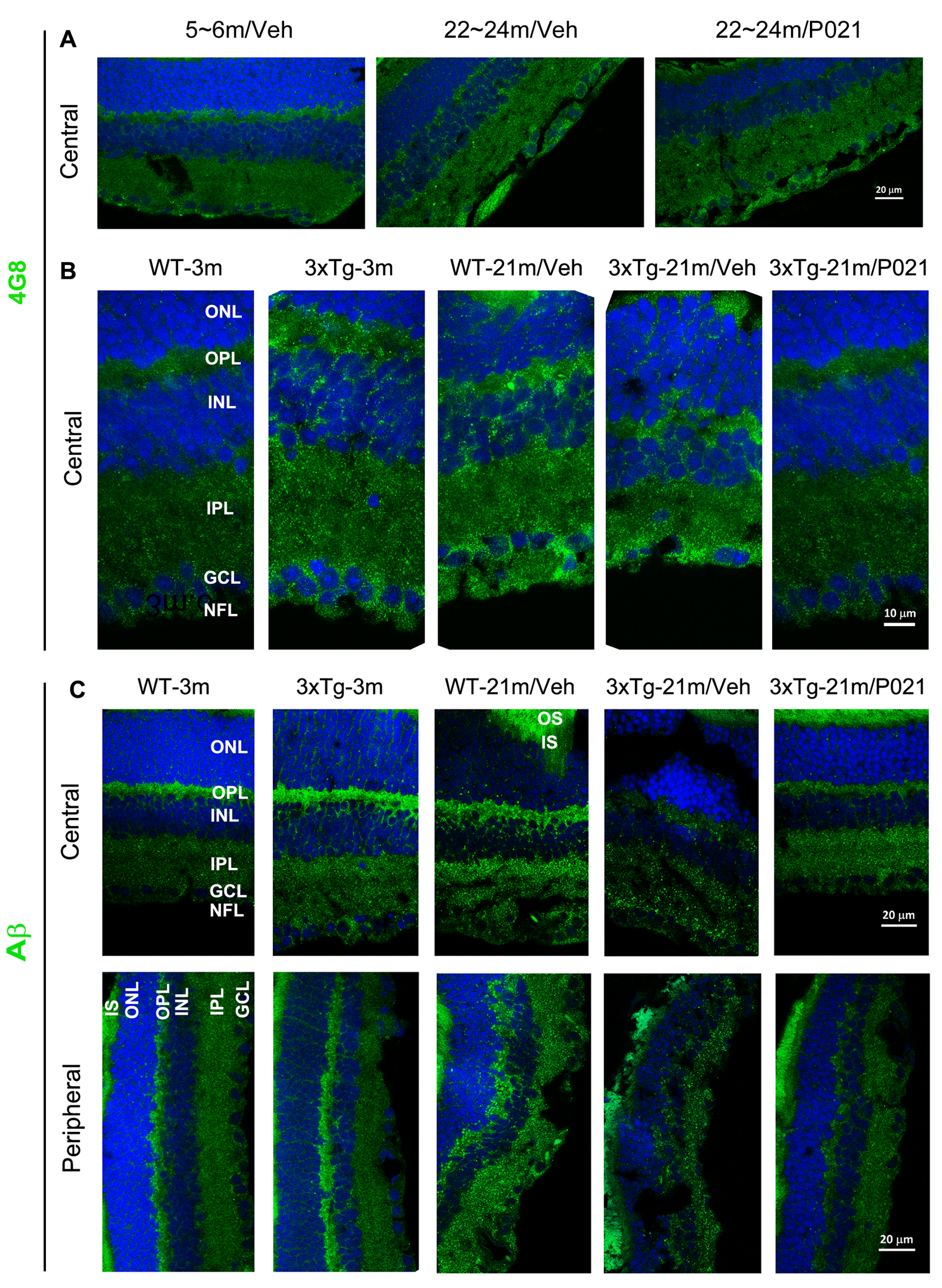


Fig. S5


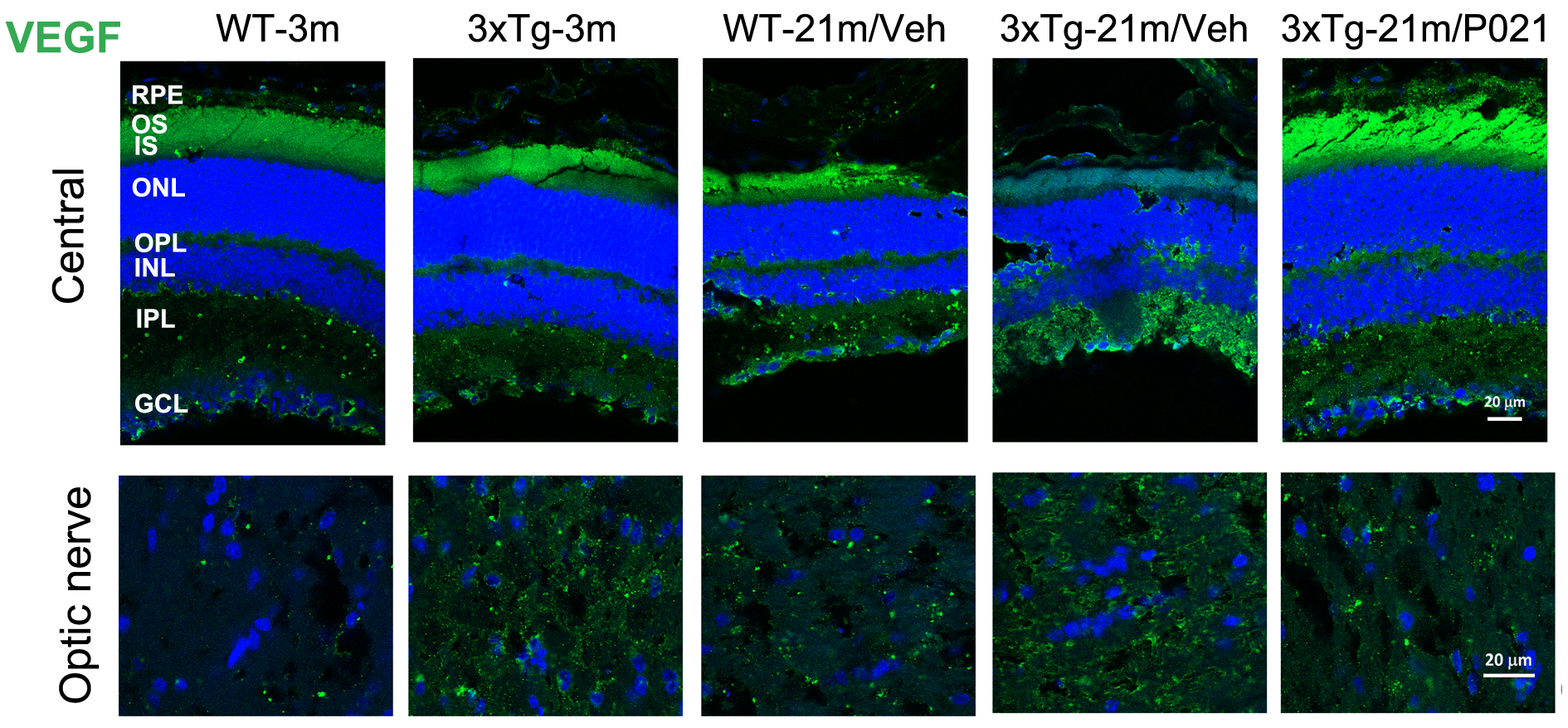


Fig. S6
